# Supplementary material for: Competition contributes to both warm and cool range edges
Source: Nat Commun. 2022 May 6;13:2502. doi: 10.1038/s41467-022-30013-3 (PMC9076896; doi:10.1038/s41467-022-30013-3)
Supplement: Supplementary file 3 — Description of Additional Supplementary Information [file 41467_2022_30013_MOESM3_ESM.pdf]

Description of Additional Supplementary Files

Title: Supplementary Data 1

Description: Regression models used to estimate focal plant size. The models were fitted using linear models. The morphological traits we measured include the number and length of flowering stems and leaves or tillers. “Stem” indicates the total length of stems of an individual calculated as the longest stem length × total stem number; “Leaf” is calculated as the longest leaf length × total leaf number; “Tiller” is calculated as the longest tiller length × total tiller number. We also used the number of leaves as a separate variable for some species (“leaf.number”). Separate regression models were fitted to predict seedling size (“seedling”; when focal plants were initially transplanted) and adult size (“adult”; when focal plants were measured at the end of the growing season) using plant samples collected in the greenhouse and field, respectively. The “seedling” model was fitted with leaf measurements, while the “seedling_leaf.number” model was only based on leaf number because leaf length data on seedlings transplanted in autumn 2017 were not available. The “adult” plant models were based on stem and leaf or tiller traits, depending on the species. When stem data of non-flowering adult plants, or leaf or tiller data on senescing plants, were not available, their size were estimated by “adult_no.stem” and “adult_stem.only” models, respectively. The number of samples (N), values of estimates (i.e., slopes) of trait predictors and R^2 values are reported. NA indicates that a morphological trait was not included in the model. See Supplementary Table 2 for species codes.

Title: Supplementary Data 2.

Description: Regression models used to predict number of seeds produced by reproductive focal plants. The models were fitted using linear models. We used zero-intercept model to avoid predictions of negative seed numbers, except for species for which the non-zero intercept models were visually determined to provide a better fit. For species whose seed number was poorly correlated with fruit length, we used an intercept-only model (i.e., mean value whose fruit length and its square are NA). See Supplementary Table 2 for species codes.

Title: Supplementary Data 3.

Description: Final models of each vital rate function for each species used to parameterize the IPM models. Shown for each model are number of samples (*N*), the Akaike information criterion corrected for small samples (AICc), the difference in AICc compared to the lowest-AICc model (ΔAICc) and the AICc weight (*w*AICc = model probability). Models with ΔAICc = 0 are the lowest-AICc models. Models in bold were not the lowest-AICc models, and in these cases additional explanation is provided. See Supplementary Table 2 for species codes.

Title: Supplementary Data 4.

Description: Complete table of vital rate model selection. The vital rates include individuals’ survival, growth, flowering, fecundity, seed germination, seedling establishment in the absence of neighbours, and seedling establishment in the presence of neighbours. The full models of size-dependent vital rates (survival, growth, flowering and fecundity) included size z at time t (size.autumn0), experimental site and competitor species (background.species) and all two- and three-way interactions, while the full models of size-independent vital rates (i.e., seed germination and seedling establishment) included only site, competitor species and their interaction. Model “1” indicates an intercept-only model (i.e., species’ mean across the three sites was used in the model). Included are the number of samples (*N*), Akaike information criterion of each model corrected for small samples (AICc), changes

in AICc compared to the lowest-AICc model (ΔAICc) and the AICc weight (*w*AICc = model probability). See Supplementary Table 2 for species codes.

Title: Supplementary Data 5.

Description: Estimated population growth rates. The estimates of population growth rates (λ) of each focal species when neighbours are absent (i.e., intrinsic growth rates when background species is “none”) and present (i.e., invasion growth rates with background species indicating the identity of the competitor) in the three study sites.

Title: Supplementary Data 6.

Description: Estimated niche differences, relative fitness differences, coexistence metrics, and competitive outcomes.
