# Supplementary material for: Competition contributes to both warm and cool range edges
Source: Nat Commun. 2022 May 6;13:2502. doi: 10.1038/s41467-022-30013-3 (PMC9076896; doi:10.1038/s41467-022-30013-3)
Supplement: Supplementary file 10 — Reporting Summary [file 41467_2022_30013_MOESM10_ESM.pdf]

nature portfolio

Corresponding author(s): Shengman Lyu

Last updated by author(s): Mar 29, 2022

# Reporting Summary

Nature Portfolio wishes to improve the reproducibility of the work that we publish. This form provides structure for consistency and transparency in reporting. For further information on Nature Portfolio policies, see our Editorial Policies and the Editorial Policy Checklist.

## Statistics

For all statistical analyses, confirm that the following items are present in the figure legend, table legend, main text, or Methods section.

| n/a | Confirmed | |
|---|---|---|
| ☐ | ☒ | The exact sample size ($n$) for each experimental group/condition, given as a discrete number and unit of measurement |
| ☐ | ☒ | A statement on whether measurements were taken from distinct samples or whether the same sample was measured repeatedly |
| ☐ | ☒ | The statistical test(s) used AND whether they are one- or two-sided<br>*Only common tests should be described solely by name; describe more complex techniques in the Methods section.* |
| ☐ | ☒ | A description of all covariates tested |
| ☐ | ☒ | A description of any assumptions or corrections, such as tests of normality and adjustment for multiple comparisons |
| ☐ | ☒ | A full description of the statistical parameters including central tendency (e.g. means) or other basic estimates (e.g. regression coefficient) AND variation (e.g. standard deviation) or associated estimates of uncertainty (e.g. confidence intervals) |
| ☐ | ☒ | For null hypothesis testing, the test statistic (e.g. $F$, $t$, $r$) with confidence intervals, effect sizes, degrees of freedom and $P$ value noted<br>*Give P values as exact values whenever suitable.* |
| ☒ | ☐ | For Bayesian analysis, information on the choice of priors and Markov chain Monte Carlo settings |
| ☒ | ☐ | For hierarchical and complex designs, identification of the appropriate level for tests and full reporting of outcomes |
| ☒ | ☐ | Estimates of effect sizes (e.g. Cohen's $d$, Pearson's $r$), indicating how they were calculated |

*Our web collection on statistics for biologists contains articles on many of the points above.*

## Software and code

Policy information about availability of computer code

| | |
|---|---|
| Data collection | No software was used for data collection. |
| Data analysis | All population modeling and statistical analyses were conducted using R version 4.0.3. All R scripts used for modelling and statistical analyses are available on GitHub: https://github.com/ShengmanLyu/competition-contributes-to-range-limits and also deposited in Zenodo: https://doi.org/10.5281/zenodo.6395222 |

For manuscripts utilizing custom algorithms or software that are central to the research but not yet described in published literature, software must be made available to editors and reviewers. We strongly encourage code deposition in a community repository (e.g. GitHub). See the Nature Portfolio guidelines for submitting code & software for further information.

## Data

Policy information about availability of data

All manuscripts must include a data availability statement. This statement should provide the following information, where applicable:

- Accession codes, unique identifiers, or web links for publicly available datasets
- A description of any restrictions on data availability
- For clinical datasets or third party data, please ensure that the statement adheres to our policy

The data we used to estimate species elevation distributions are based on a published study (see ref. 45 in the main text) and could be obtained from the author on request. The functional trait data used for species selection are obtained from the LEDA dataset (see ref. 46 in the main text) and available at https://uol.de/en/landeco/research/leda/data-files. The source data underlying Figs. 1–3 are provided as a Source Data file. The data presented in this study are available in Figshare (see ref. 53 in the main text), at https://doi.org/10.6084/m9.figshare.19108127.v1

# Field-specific reporting

Please select the one below that is the best fit for your research. If you are not sure, read the appropriate sections before making your selection.

☐ Life sciences ☐ Behavioural & social sciences ☒ Ecological, evolutionary & environmental sciences

For a reference copy of the document with all sections, see nature.com/documents/nr-reporting-summary-flat.pdf

# Ecological, evolutionary & environmental sciences study design

All studies must disclose on these points even when the disclosure is negative.

| | |
|---|---|
| Study description | We designed a field experiment to study the effects of elevation on species' population growth and competitive outcomes by growing focal plants either without competition or competing with a background monoculture of the same or another species. The full design included 56 unique interspecific pairs in each site accounting for 61% of all 14 × 13 = 91 possible pairwise combinations. These pairs were selected to evenly sample differences in functional trait space based on a pilot analysis using plant height, specific leaf area and seed mass obtained from the LEDA dataset. Each focal species competed against four lowland and four highland species, yielding 14 lowland-lowland and highland-highland pairs and 28 lowland-highland pairs. Across all three sites, this design resulted in N = 3780 individuals in total ([56 interspecific pairs × 2 + 14 intraspecific pairs + 14 non-competition] × 9 individuals × 3 sites). |
| Research sample | We included 14 herbaceous focal species that frequently occur in this region, half of which originated from low elevation (hereafter lowland species) and half from high elevation (highland species). Lowland species had upper range limits (defined as the 90th percentile of their elevation distribution) below 1500 m (with the exception of Plantago lanceolata, with a 90th percentile of 1657 m), while highland species had lower range limits (defined as the 10th percentile of their elevation distribution) above 1500 m, based on a dataset of 550 vegetation plots from the study area 45. These species consist of 12 perennial and two biennial species, which are the dominant life histories in this region. Species were selected to include a range of functional types (7 forbs, 4 grasses, 3 legumes) and functional traits (based on plant height, specific leaf area and seed mass). Seeds were obtained from regional suppliers given the large quantities that were needed to establish the experiment. See Table S2 in the manuscript for the names of plant species and seed suppliers. |
| Sampling strategy | We collected the demographic data by following all focal plants for three annual transitions (2017-2018, 2018-2019 and 2019-2020), yielding in total 729 data points per species in the final dataset. The sample size was determined to be sufficient to fit vital rate models and estimate population growth rates based on previous studies. Plant samples that were used for fitting regression models to estimate plant size fecundity were randomly selected within the background monocultures. We collected 10 samples per species per site that was sufficient to fit the regression models. |
| Data collection | We followed each focal individual between 2017 and 2020 to monitor individual-based demographic performance (i.e. vital rates). Survival was monitored twice a year at the beginning and the end of the growing season. Towards the end of the growing season in each year (August-September), we measured all individuals to record whether they flowered and estimate seed production on flowering individuals and plant size. We also measured size-related morphological traits on all focal individuals at each census (i.e. the number and/or length of flowering stalks, leaves or ramets, depending on species) to estimate focal plant size. We counted the number and measured the size of fruits on reproductive individuals to estimate seed production. We conducted a separate experiment to estimate the germination and recruitment of each species in each site. Plant performance was examined and plant samples were collected by Shengman Lyu, Loïc Liberati, Tim Murray, Cuicui Zhou, Yan Hess, Kai-Hsiu Chen and Megan Stamp. We measured plant height and leaf length using folding carpenter rulers. Air and soil temperature and soil water moisture were recorded using TMS-4 data loggers every 30 minutes (TOMST®, Czech Republic; https://tomst.com/web/en/). |
| Timing and spatial scale | The data collection started on the 1st of May and finished on the 30th of October between 2017 and 2020, with the dates varying between years due to interannual variation in phenology (about two-week difference between years). Vital rates were recorded once a year (except for survival that was recorded twice a year), allowing us to fit the annual transition models for each vital rate (see Methods). The three study sites are located across an elevation gradient in the Swiss Alps, spanning a great range of climatic gradients. The straight-line distance between the low and high site is about 10 km. |
| Data exclusions | We excluded i) focal plants transplanted in autumn 2019 due to extremely high mortality when fitting the vital rate models (ca. 90% died, N = 277), possibly because they were transplanted very late in the year (late October) and did not root successfully before winter coming; ii) plant samples when making the size-trait correlations in the cases where obvious errors were identified, such as biologically unrealistic stalk height or aboveground dry biomass. |
| Reproducibility | We did not attempt to repeat the experiment. Data collected in this study were ensured for enough replications and statistical powers to improve reproducibility. We also provided detailed information on the study subjects, experimental design and sampling procedure in the manuscript, which would allow others to repeat the experiment. |
| Randomization | The species and species pairs were randomly selected from candidates that met the experiment criteria. And the allocation of positions within plots and sampling tasks among field assistants are random. |
| Blinding | Blinding was not possible since the plots and plants had to be identified when measuring. To avoid possible bias between investigators, data collection tasks was randomly assigned to investigators, and all investigators followed the standard protocols to observe plants. |

Did the study involve field work? ☒ Yes ☐ No

## Field work, collection and transport

| | |
|---|---|
| Field conditions | The three study sites are located in the western Swiss Alps (Canton de Vaud, 46°10–46°30′N; 6°50′–7°10′E), with two of them below and one above the tree line. |
| Location | We conducted our experiments in three sites across the elevational gradients. The low site (46.2706°N, 7.0327°E, 890 m a.sl.) has a mean annual temperature (MAT) of 11.4°C and volumetric soil water (VSW) of 0.261; the middle site (46.2866°N, 7.1338°E, 1450 m a.s.l.) has a MAT of 7.9°C and VSW of 0.410; the high site (46.2875°N, 7.1687°E, 1910 m a.s.l.) has a MAT of 5.9°C and VSW of 0.438, as measured between 2019 and 2020. |
| Access & import/export | The low site was located to a pasture belonging to a local farmer and the two higher sites are located in pastures and we got permits to access from the local community. The three sites site were accessible by car and public transport. Seeds were provided by local commercial suppliers (detailed information has been included in the man text). |
| Disturbance | The experiment occupied a 10 x 10 m area at each site. We mowed grassland surrounding plots two or three times each year to prevent them from growing or dispersing seeds into the plots. At the end of the experiment, we removed all plant materials within plots and revegetate them with mixed seeds of local species. |

# Reporting for specific materials, systems and methods

We require information from authors about some types of materials, experimental systems and methods used in many studies. Here, indicate whether each material, system or method listed is relevant to your study. If you are not sure if a list item applies to your research, read the appropriate section before selecting a response.

### Materials & experimental systems

| n/a | Involved in the study | |
|---|---|---|
| ☒ | ☐ | Antibodies |
| ☒ | ☐ | Eukaryotic cell lines |
| ☒ | ☐ | Palaeontology and archaeology |
| ☒ | ☐ | Animals and other organisms |
| ☒ | ☐ | Human research participants |
| ☒ | ☐ | Clinical data |
| ☒ | ☐ | Dual use research of concern |

### Methods

| n/a | Involved in the study | |
|---|---|---|
| ☒ | ☐ | ChIP-seq |
| ☒ | ☐ | Flow cytometry |
| ☒ | ☐ | MRI-based neuroimaging |
